# A comprehensive epigenomic analysis of phenotypically distinguishable, genetically identical female and male *Daphnia pulex*

**DOI:** 10.1186/s12864-019-6415-5

**Published:** 2020-01-06

**Authors:** Jouni Kvist, Camila Gonçalves Athanàsio, Michael E. Pfrender, James B. Brown, John K. Colbourne, Leda Mirbahai

**Affiliations:** 1 Research Program for Molecular Neurology, University of Helsinki, Helsinki, Finland; 2 Department of Natural Resource Sciences, McGill University, Montréal, Quebec, Canada; 3 Department of Biological Sciences and Environmental Change Initiative, University of Notre Dame, Notre Dame, USA; 4 Environmental Genomics and Systems Biology, Lawrence Berkeley National Laboratory, Berkeley, USA; 5 Centre for Computational Biology (CCB), University of Birmingham, Birmingham, UK; 6 School of Biosciences, University of Birmingham, Birmingham, UK; 7 Warwick Medical School, University of Warwick, Coventry, UK

**Keywords:** Epigenetics, Gene expression, Evolution, Non-conventional model organisms

## Abstract

**Background:**

*Daphnia* species reproduce by cyclic parthenogenesis involving both sexual and asexual reproduction. The sex of the offspring is environmentally determined and mediated via endocrine signalling by the mother. Interestingly, male and female *Daphnia* can be genetically identical, yet display large differences in behaviour, morphology, lifespan and metabolic activity. Our goal was to integrate multiple omics datasets, including gene expression, splicing, histone modification and DNA methylation data generated from genetically identical female and male *Daphnia pulex* under controlled laboratory settings with the aim of achieving a better understanding of the underlying epigenetic factors that may contribute to the phenotypic differences observed between the two genders.

**Results:**

In this study we demonstrate that gene expression level is positively correlated with increased DNA methylation, and histone H3 trimethylation at lysine 4 (H3K4me3) at predicted promoter regions. Conversely, elevated histone H3 trimethylation at lysine 27 (H3K27me3), distributed across the entire transcript length, is negatively correlated with gene expression level. Interestingly, male *Daphnia* are dominated with epigenetic modifications that globally promote elevated gene expression, while female *Daphnia* are dominated with epigenetic modifications that reduce gene expression globally. For examples, CpG methylation (positively correlated with gene expression level) is significantly higher in almost all differentially methylated sites in male compared to female *Daphnia*. Furthermore, H3K4me3 modifications are higher in male compared to female *Daphnia* in more than 3/4 of the differentially regulated promoters. On the other hand, H3K27me3 is higher in female compared to male *Daphnia* in more than 5/6 of differentially modified sites. However, both sexes demonstrate roughly equal number of genes that are up-regulated in one gender compared to the other sex. Since, gene expression analyses typically assume that most genes are expressed at equal level among samples and different conditions, and thus cannot detect global changes affecting most genes.

**Conclusions:**

The epigenetic differences between male and female in *Daphnia pulex* are vast and dominated by changes that promote elevated gene expression in male *Daphnia*. Furthermore, the differences observed in both gene expression changes and epigenetic modifications between the genders relate to pathways that are physiologically relevant to the observed phenotypic differences.

## Background

*Daphnia* (Crustacea: Cladocera) are fresh-water branchiopods, recognized as a model organisms by the U.S. National Institutes of Health [1]. *Daphnia* are used as a model organism in various fields of research, including ecotoxicology, ecology, population genetics and molecular studies [2–5]. Species of *Daphnia* typically reproduce by cyclical parthenogenesis. During the asexual phase female *Daphnia* produce genetically identical offspring [6]. When environmental conditions deteriorate (due to crowding, shortage of food or change in day-light cycle and temperature), *Daphnia* can switch to sexual reproduction, where female *Daphnia* produce both male and female offspring [7–11]. The female *Daphnia* produce haploid eggs which are fertilized by the male during mating to form diapausing resting eggs contained in an ephippium. These resting eggs can lay dormant in the sediment for prolonged periods of time, and hatch when environmental conditions improve [12–14].

The male and female offspring produced during the sexual reproduction are genetically identical in *Daphnia* [6], with sex being determined entirely by environmental factors, a system known as environmental sex determination (ESD). *Daphnia* offers unique opportunities in studying ESD, because the parthenogenetic female *Daphnia* can be maintained indefinitely in laboratory conditions via ameiotic reproduction to form clonal lineages and subjected to experimental manipulation [1]. The switch to male production can be manipulated either by altering the environment [11] or by administering methyl farnesoate (MF) or some other juvenile hormone analog [15, 16].

The genetically identical male and female *Daphnia* have a variety of morphological and behavioural differences, including lipid metabolism, mortality, and body size [17–23]. Previous studies have investigated gene expression differences between female and male *Daphnia* in several species [1, 24–26]. Despite differences in analysis techniques and quality of reference genomes these studies have identified substantial overlap in genes with sex-biased expression [26]. In this study, our aim was to further expand our understanding of the molecular differences between genetically identical female and male *Daphnia* that show clear phenotypic differences. Epigenetic factors, are known to contribute to phenotypic diversity in the absent of genetic differences [27, 28]. Therefore, we compared whole genome bisulphite sequencing (WGBS) data, histone modification data (H3K4me3 and H3K27me3) from chromatin immunoprecipitation sequencing, splicing and gene expression data collected from female and male *Daphnia pulex* under laboratory conditions.

Previous research of DNA methylation has shown that CpG-methylation is conserved among *Daphnia* species [29, 30]. We have also shown that in *Daphnia* and other arthropods high levels of DNA methylation within gene bodies as significantly correlated with elevated gene expression levels [30]. Since all of the previous studies on DNA methylation were carried out in female *Daphnia*, we wanted to see if DNA methylation was also conserved in male *Daphnia*, or if sex-specific differences could be observed, with correlated changes in gene expression and possibly alternative splicing. The application of ChIP-seq to study histone modifications (H3K4me3 and H3K27me3) is novel for *Daphnia*, but immunological studies have demonstrated that histone modifications do occur non-uniformly in *Daphnia* and are altered during development [31, 32].

This is the first comprehensive study that combines multiple epigenomics data with the aim of achieving a comprehensive understanding of the epigenetic differences between female and male *Daphnia* with environmental sex determination. Our data provides strong evidence that epigenetic markers are differently distributed between the two genders. Furthermore, it provides evidence in support of the hypothesis that epigenetic modifications may contribute towards an overall higher expression of majority of genes in male *Daphnia* compared to female *Daphnia* and this higher overall expression of genes in male *Daphnia* may contribute and explain some of the phenotypic differences observed between the two genders.

## Results

A multiomics approach was used to characterise the molecular profile of genetically identical female and male *Daphnia pulex* Eloise Butler strain. The aim of this study was to achieve a better understanding of sex-dependent molecular differences between genetically identical female and male *D. pulex*. To achieve this goal, the omics data (gene expression, ChIP-seq, DNA methylation and splicing data) were analysed both individually and in association with each other. This study provides the first insight into epigenetic and transcriptional differences between genetically identical genders of the model organism *Daphnia* that have evolved distinct morphological, physiological and behavioural differences.

### *Gene expression changes between male and female D. pulex*

**Fig. 1:**
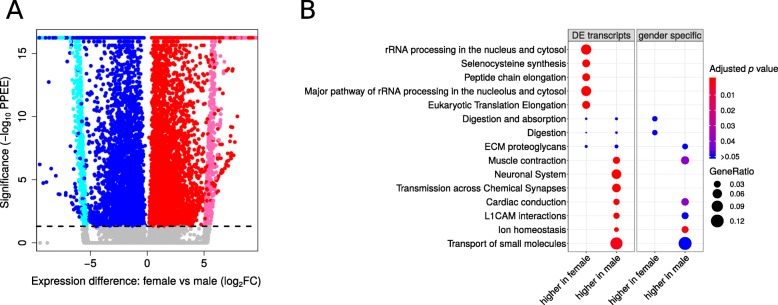
Differentially expressed transcripts between male and female *Daphnia pulex* (EB45) **a**) Volcano plot of differentially expressed transcripts. The transcripts marked with color are significantly different (Posterior Probablity of Equivalent Expression; PPEE < 0.05) between the sexes, (red = higher expression in female, blue = higher expression in male, pink = only expressed in female, light blue = only expressed in male). **b**) Reactome enrichment analysis for differentially (PPEE< 0.05) expressed transcripts. The enrichment analysis is carried out separately for the transcripts that have higher expression in male or female as well as for transcripts that are uniquely expression in one gender

We analysed expression differences between male and female *Daphnia pulex* at the transcriptome and gene level. A significant expression difference (with Posterior Probability of Equivalent Expression: PPEE< 0.05) was observed in 11.2% (12,266/109,840) of the transcripts, which originate from 23.6% (7830/33,139) of the genes. The expression differences are symmetrically distributed, except for a slight excess of transcripts (55% higher in female *Daphnia* compared to 45% higher in male *Daphnia*) with higher expression in female *Daphnia* (Fig. 1a; Additional file 1: Table S1A).

The transcripts with higher expression in female *Daphnia* are enriched for RNA processing pathways (in particular rRNA) and translation, while the transcripts with higher expression in male *Daphnia* are enriched for muscle contraction, cardiac conduction, neuronal systems and cell signalling (Additional file 2: Table S2A). A small subset (13%) of transcripts (1614 transcripts in 1313 genes) are exclusively expressed in one gender. Half of these (805 transcript) are male specific (not expressed in female *Daphnia*), and half are female specific (809 transcripts; Fig. 1a). The transcripts that are uniquely expressed in female are not significantly enriched, and the male specific transcripts are enriched for the same pathways identified for the full set of differentially expressed transcripts (Additional file 2: Table S2A; Fig. 1b).

Most of the genes with differentially expressed transcripts were also differentially expressed when analysed at the gene level (71%; 5553/7830; Additional file 1: Table S1B), while a small subset of genes were differentially expressed only at the transcript level (either alternative splicing, alternative start or stop site usage) (Additional file 1: Table S1A; Additional file 1: Table S1B). The genes with only transcript level differences were enriched for the same pathways identified for the full set of differentially expressed transcripts (including RNA processing, muscle contraction and cell-cell communication; Additional file 2: Table S2A – S2C).

We detected 3291 potential splicing events using KisSplice (Additional file 1: Table S1C). The most common splicing event was intron retention (1244), followed by alternative acceptor and/or donator site usage (1142), with exon skipping being the third most common type (524). Very few splicing events (284) were significantly (FDR < 0.05) altered between male and female *Daphnia*. The splicing types were the same for the sex-specific events and all detected splicing events (chi-squared = 80, *p* value = 0.24), and they occurred mostly in the same genes that were already identified as having differentially expressed transcripts (80%; 226/284). The genes with detected sex-specific splicing changes were not significantly enriched for Reactome pathways (Additional file 2: Table S2D).

### *DNA methylation changes between male and female D. pulex*

**Fig. 2:**
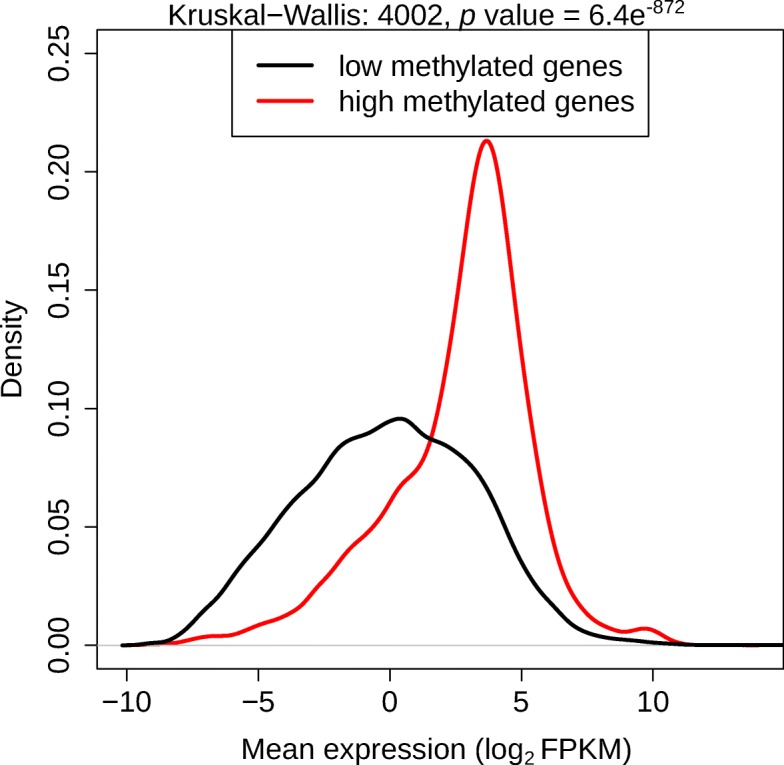
Density plot of mean expression levels (log_2_ FPKM) for genes that contain high methylated CpGs (> 50% median methylation; 2747 genes) and low methylated CpGs (< 50% median methylation; 33,139 genes) within exons 2–4 in the primary transcript in *Daphnia pulex* Eloise Butler (EB45)

We performed whole genome bisulfite sequencing (WGBS) of *Daphnia pulex* Eloise Butler strain (genotypes EB31 and EB45). We quantified the methylation level of individual CpG sites (the ratio of methylated reads to read coverage at each site). The majority of CpG sites in *Daphnia* are unmethylated or have extremely low methylation level [29, 30, 33]. The high methylated CpGs (with median methylation level > 50%) are located mostly within exons (83%; 10,599/12,790 CpGs). Almost all of these (94.5%) are within the first four exons (with 1803, 4278, 2901 and 1074 CpGs in exons 1–4 respectively) in the primary transcripts, with exon 2 having the highest occurrence (40.4%) of high methylated CpGs. The primary transcripts containing high methylated CpGs (within exons 2–4) also have substantially higher expression level compared to the transcripts with only low methylated CpGs (Fig. 2).

**Fig. 3:**
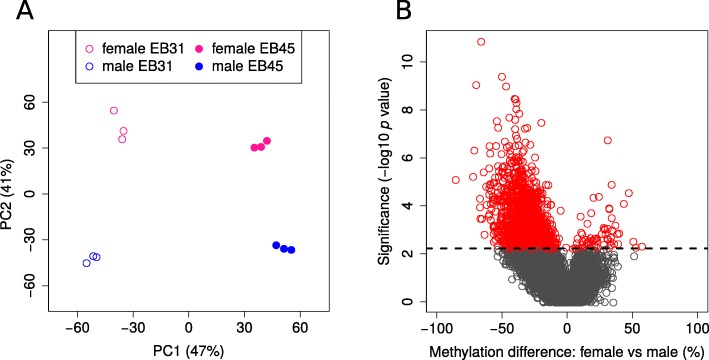
DNA methylation differences between male and female *Daphnia pulex* in Eloise Butler strain (genotypes EB31 and EB45), using a filtered dataset; CpGs not covered in all samples and with no methylated reads in more than half of the samples were excluded. **a**) Principal component analysis (PCA) of DNA methylation (CpG) levels. Samples are represented by points along PC1 (x-axis) and PC2 (y-axis), which account for the majority of variance in the data. Genotypes separate by PC1 which accounts for 47% of the variance in methylation and the sexes separate by PC2 which accounts for 41% of variance. **b**) Volcano plot of DNA methylation (CpG) differences between male and female. The differentially methylated CpGs (DMCs; FDR < 0.05) are indicated in red

After filtering out CpG sites with no methylated reads in more than half of the samples, only 18,951 sites remained for further analysis. The variation among the samples in the filtered CpG sites could be primarily attributed to differences between genotypes (EB45 vs EB31; PC1: 47% of variation) and sexes (female vs male; PC2: 41% of variation) (Fig. 3a). The CpG methylation level in male samples is globally higher than in female samples, with more than 70% of all CpGs having higher methylation level in male compared to female samples (Fig. 3b). A statistically significant difference in the methylation levels in CpG sites (FDR < 0.05) was observed for 1841 CpGs (9.71%) between male and female *Daphnia* (Additional file 1: Table S1D). The differentially methylated CpGs (DMC) are located within gene bodies (97.56%; 1796/1841), and in particular within the first four exons (78.67%; 1413/1796). Very few DMCs are located outside of known genes (2.4%; 45/1841) (Additional file 1: Table S1D) and almost all of the DMCs have higher methylation level in male *Daphnia* (96.46%, 1776/1841 DMCs) compared to female *Daphnia* (Fig. 3b).

**Fig. 4:**
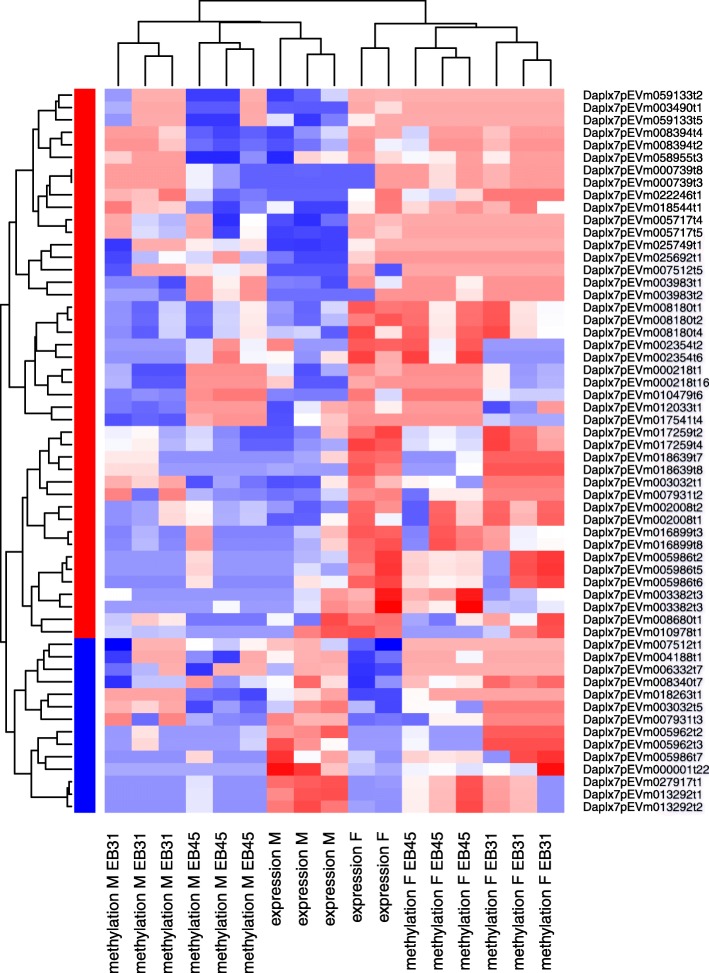
Heatmap of expression and DNA methylation for the transcripts that contain differentially methylated CpGs, where the methylation is significantly lower in male compared to female *Daphnia*. The expression and methylation levels were scaled from 0 to 1, with red indicating high expression or high methylation and blue low expression or low methylation. The sidebar shows the average direction of expression change, with red indicating increased expression and blue decreased expression in female compared to male *Daphnia*

The DMCs with higher methylation in male *Daphnia* are not significantly enriched for any known pathways (Additional file 2: Table S2E). This potentially indicates that the higher methylation of genes in male *Daphnia* compared to female *Daphnia* is non-specific and global. The few genes with lower methylation level in male *Daphnia* compared to female *Daphnia* are however enriched for specific cellular functions, including cellular senescence, interleukin-17 signalling and negative regulation of FGFR signalling (Additional file 2: Table S2E). The transcripts containing DMC with decreased methylation in male *Daphnia* also demonstrate a reduced expression compared to female *Daphnia* for ~ 80% of the transcripts (Fig. 4), while the DMCs with increased methylation in male *Daphnia* have no association with expression level at the transcript level.

### *Histone modification changes between male and female D. pulex*

The initial ChIP-peaks identified with MACS2 are substantially smaller for histone H3 trimethylated at lysine 27 (H3K27me3; with a median size of 318 bp) compared to histone H3 trimethylated at lysine 4 (H3K4me3; 800 bp). During the differential peak analysis (DiffBind) overlapping peaks are merged resulting in slightly larger peaks (488 bp for H3K27me3 and 968 bp for H3K4me3). The H3K4me3 peaks are more sparsely located in the genome with a 3089 bp median distance between peaks, compared to 430 bp for H3K27me3 (with long stretches of nearby peaks). The peak intensity (ChIP compared to input controls) for H3K4me3 is higher than for H3K27me3, with a median fold change of 5.15 vs 2.02 in the initial peak discovery, and 7.08 vs 4.95 in the differential peak analysis for H3K4me3 and H3K27me3, respectively (Additional file 1: Table S1E; Additional file 1: Table S1F).

**Fig. 5:**
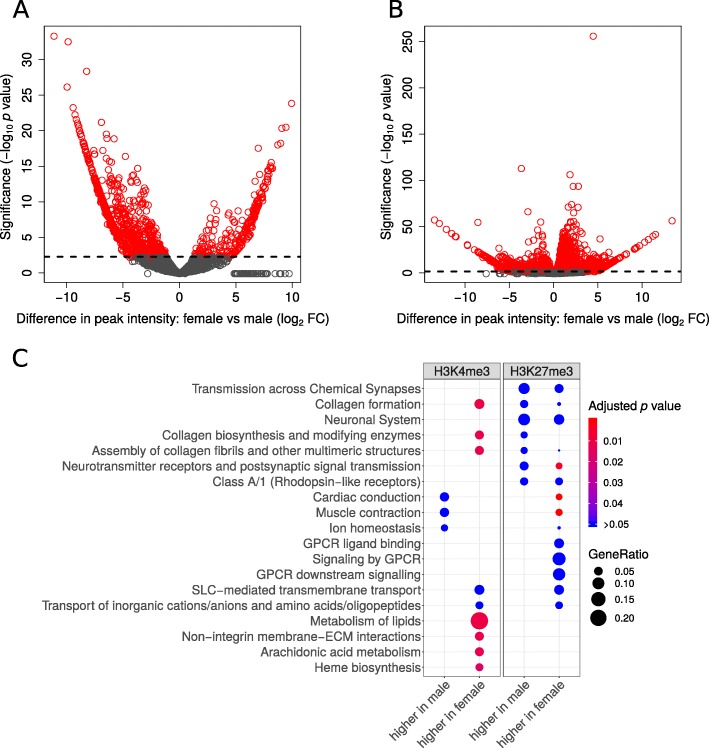
Differentially regulated histone modifications between male and female *Daphnia pulex*. A) Volcano plot for H3K4me3, B) Volcano plot for H3K27me3 between male and female. The differentially modified histone peaks (FDR < 0.05) are indicated in red. C) Reactome enrichment analysis of differential histone modifications analysed separately for transcripts that have higher peak intensity in male or female

We identified 10,092 H3K4me3 peaks, 95% (9602) of which are consistently found (FDR < 0.05) in all samples (*n* = 6) compared to input controls (*n* = 2) (Additional file 1: Table S1E). Almost all (97%; 9365) of these peaks are within 200 bp of known genes (10,968 genes, with some peaks overlapping with more than one gene), and enriched at the start of the gene, with 90% (8438) overlapping with exon 1. About 10% (1061) of the H3K4me3 peaks have sex-specific differences in intensity (FDR < 0.05), with 78% (833) of the sex-specific peaks having higher intensity in male *Daphnia* (in 1068 genes) and 22% (228) having higher intensity in female *Daphnia* (in 275 genes) (Fig. 5a). The genes with higher H3K4me3 intensity in female *Daphnia* compared to male *Daphnia* are enriched for multiple Reactome pathways, including collagen formation, lipid metabolism, heme biosynthesis, extracellular matrix organization and cell motility via c-Met signalling pathway. Whereas the genes with higher H3K4me3 intensity in male *Daphnia* are only marginally enriched for cardiac conduction and related pathways (Fig. 5c; Additional file 2: Table S2F).

We identified almost three times as many peaks (29,162) for H3K27me3, compared to H3K4me3. Similar to H3K4me3, most of the peaks (97%) are consistently found (28,372/29,162; FDR < 0.05) in all samples compared to the input controls, and are associated (99%; 28,284 peaks) with known genes (12,901 genes; Additional file 1: Table S1F). Overall, 41% (12,102) of the H3K27me3 peaks (in 7329 genes) had different intensities (FDR < 0.05) between male and female *Daphnia*. In contrast to the gene expression promoting H3K4me3 histone modification, the expression suppressing H3K27me3 modification occurred predominantly (> 86%; 10,356) in female *Daphnia* (in 6123 genes), while only 14% (1753) of the H3K27me3 peaks had higher intensity in male *Daphnia* (in 1296 genes) (Fig. 5b). The genes with higher H3K27me3 intensity in female compared to male *Daphnia* are enriched for multiple Reactome pathways including GPCR signalling, transport of small molecules, G-Protein alpha-i signalling, digestion, muscle contraction and neuronal systems. Whereas the genes with higher H3K27me3 intensity in male *Daphnia* are not significantly enriched for any Reactome pathways (Fig. 5c; Additional file 2: Table S2G).

**Fig. 6:**
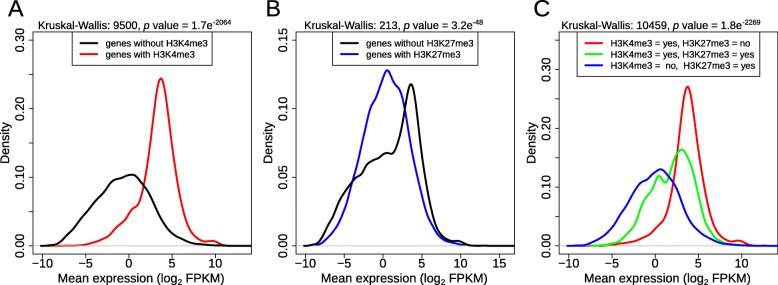
Expression densities for genes with or without histone modifications. **a** H3K4me3, **b**) H3K27me3, **c**) both H3K4me3 and H3K27me3. The expression level (FPKM) is averaged across all samples and log_2_-tranformed.

The histone modifications have significant association with gene expression. Genes with H3K4me3 modifications have two times higher mean expression (FPKM 31.97 vs 15.95), compared to genes without H3K4me3 modifications (Fig. 6a). The opposite pattern is observed for H3K27me3 modifications. Genes with H3K27me3 modifications have two times lower mean expression (FPKM 14.20 vs 24.28), compared to genes without H3K27me3 modifications (Fig. 6b). While genes containing both modifications have an intermediate expression level (Fig. 6c).

### Integrative analysis: covariation and co-occurrence

**Fig. 7:**
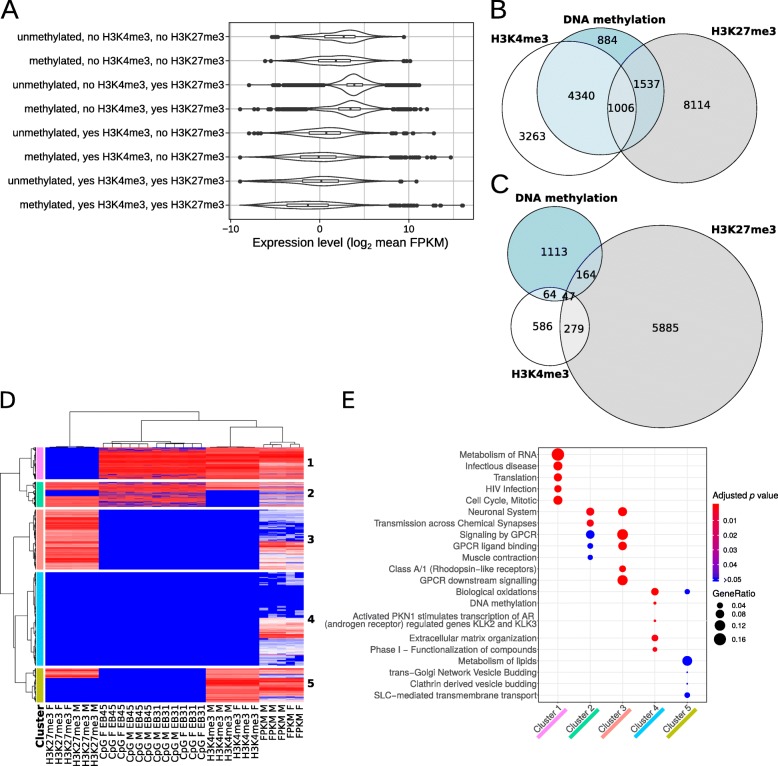
Combined comparison of DNA methylation, histone modifications and gene expression. **a** Violin plot of gene expression separated by presence/absence of DNA methylation and histone modifications: H3K4me3 and H3K27me3. The average gene expression in all samples, Fragments Per Kilobase of transcript per Million (FPKM) is log_2_ scaled. **b** Venn diagram of genes with DNA methylation and histone modifications, for all genes with detectable modifications above the filtering cutoffs specified in the methods. **c** Venn diagram for genes, with significant differences (FDR < 0.05) between male and female *Daphnia pulex* for the modifications. **d** Heatmap of ranked values for gene expression (FPKM), histone modifications (H3K4me3 and H3K27me3), and DNA methylation (CpG). Red indicates high level of expression or modification, blue indicates low level of expression or modifications. Genes separate into 5 main clusters by omics profile. **e** Enrichment results for the most significant Reactome pathways in the main clusters from the heatmap (1–5)

The DNA methylation and histone modifications affect gene expression in an additive manner (Fig. 7a). DNA methylation (in exons) increases gene expression (from mean FPKM 18.17 to 32.21) regardless of histone modifications. The presence of H3K4me3 in methylated genes additionally increase expression (to FPKM 40.25), while H3K27me3 decreases expression (to FPKM 11.62). The highest expression is observed in genes that have both DNA methylation, contain H3K4me3 and are absent of H3K27me3 modifications (mean FPKM 41.59). While the lowest expressed genes are absent of all modifications. The very low expressed genes undoubtedly contain genes with mapping problems (highly variable or partial genes), which could result in reduced detection in all datasets.

The majority of the genes containing DNA methylation (69.19%) also contain H3K4me3 histone modifications (chi-squared = 7615.5, *p* value = 2.9e^− 1656^), which is more than twice the value one would expect by chance (5346 genes observed with both modifications compared to 2281 genes expected by chance). While the overlap among genes with H3K27me3 and DNA methylation (obs: 2543 vs exp.: 2759; chi-squared = 34.1, *p* value = 5.2e^− 09^) or H3K27me3 and H3K4me3 (obs: 2181 vs exp.: 3493; chi-squared = 1087.1, *p* value = 2.1e^− 238^) is significantly underrepresented considering the huge number of genes containing these modifications (Fig. 7b).

While the overlap is substantially smaller for the genes where these modifications are different between male and female *Daphnia* (Fig. 7c), the overlap is still significantly different than one would expect by chance. The overlap between DNA methylation and H3K4me3 is significantly enriched (111 genes with both modifications compared to 41 expected by chance; chi-squared = 123.7, *p* value = 1.0e^− 28^) as is the overlap between H3K4me3 and H3K27me3 (obs: 326 vs exp.: 188; chi-squared = 128.9, *p* value = 7.0e^− 30^). The overlap between DNA methylation and H3K27me3 is significantly underrepresented (obs: 211 vs exp.: 271; chi-squared = 16.8, *p* value = 4.1e^− 05^).

Most genes with sex-specific differences contain a single modification, especially when contrasted to the global background of DNA methylation and histone methylations, where the overlap is substantial. The few sex-specific genes that contain multiple modifications are not significantly enriched to any particular pathway after multiple testing corrections (Additional file 2: Table S2H). Granted each of these omics methods has their own unique strengths and weakness (unique error profiles, statistical power and heterogeneity among samples), which could partially explain the low level of overlap. On the other hand, the global background of co-occurring DNA methylation and histone modifications (Fig. 7d) are significantly enriched for multiple pathways (Fig. 7e; Additional file 2: Table S2I). Many of the same pathways are enriched in the individual analyses for DNA methylation, histone modification and gene expression for the sex-specific differences (Table 1).

**Table 1:** Summary of enriched Reactome pathways across multiple omics datasets comparing male and female *Daphnia pulex*

ID	Description	DE transcripts	DNA-methylation	H3K27me3	H3K4me3
R-HSA-192823	Viral mRNA Translation	higher in female			
R-HSA-927802	Nonsense-Mediated Decay (NMD)	higher in female			
R-HSA-5578775	Ion homeostasis	higher in male ^a^			
R-HSA-72766	Translation	higher in female	higher in female		
R-HSA-156827	L13a-mediated translational silencing of Ceruloplasmin expression	higher in female	higher in female		
R-HSA-72706	GTP hydrolysis and joining of the 60S ribosomal subunit	higher in female	higher in female		
R-HSA-72613	Eukaryotic Translation Initiation	higher in female	higher in female		
R-HSA-72737	Cap-dependent Translation Initiation	higher in female	higher in female		
R-HSA-2559583	Cellular Senescence		higher in female		
R-HSA-983169	Class I MHC mediated antigen processing & presentation		higher in female		
R-HSA-189451	Heme biosynthesis				higher in female
R-HSA-8875878	MET promotes cell motility				higher in female
R-HSA-556833	Metabolism of lipids				higher in female
R-HSA-211859	Biological oxidations			higher in female	higher in female
R-HSA-1474244	Extracellular matrix organization	higher in male		higher in female	higher in female
R-HSA-1474228	Degradation of the extracellular matrix	higher in male		higher in female	higher in female
R-HSA-397014	Muscle contraction	higher in male ^a^		higher in female	
R-HSA-5576891	Cardiac conduction	higher in male ^a^		higher in female	
R-HSA-6805567	Keratinization	higher in male		higher in female	
R-HSA-6809371	Formation of the cornified envelope	higher in male		higher in female	
R-HSA-392154	Nitric oxide stimulates guanylate cyclase	higher in male		higher in female	
R-HSA-418346	Platelet homeostasis	higher in male		higher in female	
R-HSA-112316	Neuronal System	higher in male		higher in female	
R-HSA-112314	Neurotransmitter receptors and postsynaptic signal transmission	higher in male		higher in female	
R-HSA-112315	Transmission across Chemical Synapses	higher in male		higher in female	
R-HSA-382551	Transport of small molecules	higher in male		higher in female	
R-HSA-425393	Transport of inorganic cations/anions and amino acids/oligopeptides	higher in male		higher in female	
R-HSA-425407	SLC-mediated transmembrane transport	higher in male		higher in female	
R-HSA-109582	Hemostasis	higher in male		higher in female	
R-HSA-8935690	Digestion			higher in female	
R-HSA-372790	Signaling by GPCR			higher in female	

^a^ sex specific expression

**Fig. 8:**
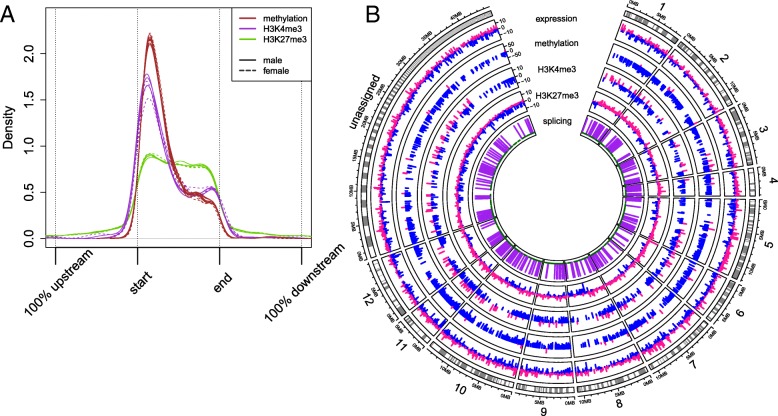
**a**) Density plot of the epigenetic modification. Showing the relative locations of histone modifications (H3K4me3 and H3K27me3) and CpG methylations (in different colour), scaled to the start and end of the primary transcript of each gene. Extremely short and long genes are excluded (transcript length below 1000 bp or above 10,000 bp). The modifications are mapped to the nearest gene, taking the relative distance to the start of the gene from the peak maximum separately for each sample (genders are indicated with line type). **b** Circos-plot of sex-specific differences in the multiple omics datasets, distributed across the genome. The scaffolds assignment to chromosomes is based on [34]. The direction of change is indicated with color; blue = higher in male, pink = higher in female. The differentially spliced genes are indicated in purple and the alternatively spliced genes that do not display sex-specific difference are indicated in green

The sex-specific changes in gene expression, DNA methylation, histone modifications and alternative splicing are evenly distributed across the genome (scaffolds assigned to chromosomes according to Ye et al. 2017) (Fig. 8), with slight excess from an expected distribution in chromosomes 9 and 11 for DNA methylation, H3K4me3 and H3K27me3 and chromosome 4 for gene expression and alternative splicing.

## Discussion

Sex determination, a fundamental biological process, impact the development of most organs and causes sex-specific differences in behaviour, physiology and morphology [35]. Sex determination in majority of organisms is underpinned and regulated either by genetic factors (GSD: genetic sex determination) or environmental factors (ESD: environmental sex determination). The latter is initiated by environmental cues, such as temperature, nutrition, population density and photoperiod. ESD is observed in range of species across the animal taxa, such as rotifers, nematodes, crustaceans, insects, fishes and reptiles [35]. The crustacean *Daphnia* are also subject to environmental sex determination [36], where by the same genotype may develop into phenotypically distinct male and female *Daphnia* depending on the environmental cues [26, 37]. The genetically identical male and female *Daphnia* demonstrate differences in their phenotype and life-history traits, including metabolic activity, mortality, morphology (antenna and carapace) and body size [17, 18]. In particular the female *Daphnia* are larger, almost twice the size of male *Daphnia*, while the male *Daphnia* have a higher metabolic rate and shorter lifespan compared to female *Daphnia* [21–23]. Once the sex is determined it is maintained throughout the life of the organism even in the absence of the initial environmental cue [11, 16]. The maintenance of the acquired gender throughout the life of an organism can be caused by early developmental changes which result in a cascade of differences including structural alterations. It can also include regulatory factors such as hormones that need to be constantly maintained at specific levels. Such regulatory factors can also include epigenetic factors that help to maintain the acquired phenotype, leading to creating a sex-specific molecular signature. Our goal in this study was to achieve a better characterisation and understanding of the sex-specific differences (signature) at a molecular level with a specific focus on contribution of epigenetic factors (histone modifications and DNA methylation). To achieve this objective, we generated omics data at multiple levels to create a molecular signature for female and male *Daphnia.*

Previous studies have investigated differences in the expression levels of genes between female and male *Daphnia* (in *D. pulex*, *D. magna* and *D. galeata)* [1, 24–26]. Our study differs from previous published work as in addition to investigate differences in expression at the gene level, we also investigated changes in expression at the transcriptome level identifying variation in alternative splicing and usage of alternative start and stop sites. Our data indicated that the genes with the same basal expression level containing differentially expressed alternative isoforms between female and male *Daphnia* were enriched for RNA processing pathways and translation regulation. However, the genes with detected splicing variations were not significantly enriched for Reactome pathways. The alternative splice variants that are differentially regulated between the sexes may represent a diverse set of tissue specific changes, in line with the morphological differences between the sexes.

Our results, similar to previous findings, demonstrate that a large portion of genes display significant differences in the expression between male and female *Daphnia*, affecting more than 1/5 of all annotated genes. We further compared our list of sex-specific genes to *D. magna* [26]. The two species *D. magna* and *D. pulex* are among the most distantly related *Daphnia* species and span the entire phylogeny of the genus, having diverged more than 200 million years ago [38]. In *D. magna*, 42% of the genes are reported as differentially expressed between male and female [26], which is substantially higher than what we detected in *D. pulex* (~ 20%). Out of the 11,197 differentially expressed genes in *D. magna*, we could find a reliable ortholog in *D. pulex* for 7920 genes (using blastp with e-value <1e^− 20^). The agreement between *D. pulex* and *D. magna* for the identified 7920 sex-specific genes is substantial. Without filtering the data based on significance, in *D. pulex* > 73% of the genes have the same direction of expression change as in *D. magna*. When selecting only the genes that we detected as significantly differentially expressed (3093/7920 genes) the agreement increases to > 86%. Not only is the direction of change the same but also the magnitude of expression change is highly correlated (R^2^ = 0.55, *p* value < 2.2e^− 16^), especially for the genes with higher expression in female *Daphnia* (Additional file 3: Fig. S1). This potentially indicates that sex-specific genes and the enriched pathways (e.g. RNA metabolisms, signalling and development) are conserved between the two species and are essential for maintaining sex-specific characteristics.

It is worth highlighting that these conserved genes included known sex-determination factors. For example, in *Daphnia* there are several orthologs for the *Drosophila doublesex* (*dsx*) gene, which are not alternatively spliced as in insects, but regulate sex determination by expression level [39]. In *Daphnia magna* two of these genes (*DapmaDsx1*: APZ42_027481, *DapmaDsx2*: APZ42_027480) have elevated expression in male *Daphnia*, with DapmaDsx1 being capable of regulating male morphology when ectopically applied and female traits when knocked-down during embryogenesis [35]. The *Daphnia pulex* orthologs of *DapmaDsx1* (Daplx7pEVm013292) and *DapmaDsx2* (Daplx7pEVm013921) both have higher expression in male *Daphnia* (log_2_FC = − 4.02 and log_2_FC = − 6.18, respectively, with PPEE< 2.2e^− 16^ for both; Additional file 3: Fig. S1), and also contain significant differences in the modification H3K4me3, with higher level in male *Daphnia* (log_2_FC = − 8.25, FDR = 1.20e^− 25^ and log_2_FC = − 4.98, FDR = 7.87e^− 06^), whereas female *Daphnia* had higher level of H3K27me3 modification in both genes (log_2_FC = 12.40, FDR = 1.79e^− 40^ and log_2_FC = 13.34, FDR = 1.87e^− 54^; Additional file 1: Table S1).

Histone modifications can rapidly regulate the expression of genes [40, 41]. In this study, we analysed two histone modifications, H3K4me3 and H3K27me3, known to regulate the expression of genes in a variety of species [42, 43]. H3K4me3 modification is a hallmark of actively transcribed genes and it is commonly associated with transcription start sites (TSS) and promoter regions [44], whereas H3K27me3 peaks at the TSS and promoter region, it is more spread out along the length of the affected genes than the H3K4me3 modification. Furthermore, H3K27me3 is strongly associated with down-regulation of nearby genes via the formation of heterochromatic regions [45]. Both active and inactive modifications can be found in *Daphnia* in the expected locations (Fig. 8a). The H3K4me3 modifications were concentrated at start of the genes, with 97% of the detected peaks within 200 bp of the known transcription start site. While H3K27me3 modifications occurred throughout the gene body and intergenic regions. The majority of the histone modification peaks were observed in both male and female *Daphnia*. The effect of the histone modifications on gene expression level was clear and in line with the expectations (Fig. 6) with H3K4me3 modification promoting higher expression level and H3K27me3 modification generally suppressing the expression of the genes. Most interestingly, we observed that the majority of sex-specific H3K4me3 peak are higher in male *Daphnia* (78%), while female *Daphnia* are dominated by higher H3K27me3 peaks (86%). This difference can potentially indicate a higher basal level of global expression in male compared to female *Daphnia*. We also detected a relatively small number of genes where both modifications were present (Fig. 7b). This resulted in an intermediate expression level (Fig.6c) potentially creating genes in a poised status ready to be either expressed or suppressed (higher expression compared to genes with only H3K27me3 and lower than genes with only H3K4me3) [46–48]. However, the latter category requires further investigation to remove the possibility of mix peak signal due to presence of multiple cell populations.

In addition to histone modifications, we investigated the differences in CpG methylation between the two genders. Similar to our previous findings, the majority of the methylated CpG sites in both genders were located within the gene body and mostly concentrated at exons 2–4 region [30]. Genes with high levels of CpG methylation (> 50%) in both genders demonstrated an elevated expression level compared to rest of the genes (Fig. 2; similar to Kvist et al., 2018). Furthermore, based on our data, the two epigenetic modifications of CpG methylation and H3K4me3 demonstrated a complementary and additive effect on gene expression. As demonstrated in Fig. 7a, genes with both modifications had a significantly higher expression level compared to the rest of the genes. Most interestingly, CpG methylation levels are overall significantly higher (96% of all DMC) in male compared to female *Daphnia*. This observed non-specific global higher level of methylation in male *Daphnia* coupled with higher H3K4me3 peaks in male compared to female *Daphnia* could further suggest a potential basal global higher gene expression in males. However, at the gene expression level there is no obvious bias in male *Daphnia* demonstrating a higher expression for majority of the genes compared to female *Daphnia*. In fact, there are slightly more genes (5% more) with higher expression in female compared to male *Daphnia*. Although our data does not support a male biased higher gene expression level, the existence of such bias in gene expression cannot be entirely ruled out at this stage as methods used for normalising the data, library preparation and RNA-seq analysis can mask global biases [49]. In order to evaluate if a global bias in gene expression truly exists between male and female *Daphnia,* one would need to use external spike-in references during sample preparation, which would tie the cell counts to mRNA yields, and permit absolute quantification of gene expression. The traditional normalization methods used (in this study and all other *Daphnia* gene expression studies) assume that most genes are expressed at the same level among samples, and cannot detect global biases that affect all or most genes [49]. An alternative explanation is that the lack of male biased gene expression level, which is observed at histone modification and CpG methylation levels, could be real. It is feasible that there are compensatory changes in female *Daphnia* (besides the ones studied here) that balance, and slightly (5% of genes) increase, the level of gene expression between female and male *Daphnia*. For example in mouse lymphocytes, elevated expression of a single transcription factor (*c-myc*) can result in a global transcriptional amplification of all actively transcribed genes [50]. The *Daphnia pulex* ortholog of *c-myc* (Daplx7pEVm006187) was indeed elevated in female *Daphnia pulex* in this study (log_2_FC = 1.39 higher in female compare to male, PPEE< 2.2e^− 16^). As well as in *D. magna* (APZ42_014785) in another study (log_2_FC = 0.64 higher expression in female compared to male, adjusted *p* value = 5.3e^− 05^) [26].

Enrichment analysis demonstrated that genes with higher CpG methylation and histone modifications in male *Daphnia* were not enriched for specific pathways and were mostly randomly distributed across the genome. In contrast, genes containing higher CpG methylation levels in female *Daphnia* were enriched for partially linked pathways related to immune response (Toll like receptor cascades, Interleukin-17 signalling, Class I MHC mediated antigen processing & presentation, and TRAF6 mediated induction of NFkB and MAP kinases upon TLR7/8 or 9 activation) and ageing (Cellular senescence, Senescence-Associated Secretory Phenotype, MAP kinase activation, and Negative regulation of FGFR signalling). Enrichment of these particular pathways in female *Daphnia* may be related to the fact that female *Daphnia* typically have a longer lifespan compared to male *Daphnia* [21–23], although few male strains maintained under specific conditions have shown to outlive females [51]. The enriched pathways could explain some of the phenotypic differences observed between female and male *Daphnia*. For examples, the heat shock response protects the cells against a plethora of external and internal damage, including elevated temperature, oxidative damage, metal stress and also ageing related protein misfolding and aggregation [52, 53]. Heat shock proteins (HSPs) can also activate innate immune system [54]. HSPs are differentially expressed between sexes in *Daphnia*, with most HSPs having higher expression in female *Daphnia*. Also HSPs react more strongly to heat stress in female *Daphnia* [55]. In comparisons among *Daphnia* species elevated HSP expression is associated with longer lifespan [56]. We observed 80% of the differentially expressed heat shock proteins (11/14 genes) having higher expression in female compared to male *Daphnia*, including heat shock transcription factor 1 (*HSF1*; Daplx7pEVm005655, log_2_FC = 0.52), despite HSF1 having (9.43%) higher methylation level in male *Daphnia*.

Male *Daphnia* grow more slowly compared to female *Daphnia* and reach a smaller body size [17, 18]. Female *Daphnia* accumulate lipids they acquire from their food [19], which are used for producing eggs (sexual and asexual) [57, 58]. These morphological differences are in line with the enrichment results for the relatively few genes that had higher H3K4me3 levels in female *Daphnia* (Metabolism of lipids, Biological oxidations and Heme biosynthesis). Male *Daphnia* are typically smaller than female *Daphnia*, are more active, and faster swimmers [20], have faster heartbeat rate [22] and in general have higher metabolic activity compared to female *Daphnia*. These differences are reflected in the patterns of gene expression with enriched pathways for muscle activity (Ion homeostasis, Muscle contraction and Cardiac conduction) for genes with higher expression in male compared to female *Daphnia* (Additional file 2: Table S2F).

## Conclusions

Overall, our study indicates that genetically identical female and male *Daphnia* have evolved distinct DNA methylation, histone modification and gene expression patterns which could explain the differences in morphology, physiology and behaviour between male and female *Daphnia*. As discussed, some of the changes observed at the gene (*doublesex* genes and HSP genes) and pathway (cellular senescence pathway and immune response) levels support this hypothesis. Furthermore, this is the first multi-omics study that provides insight into interactions between histone modifications (H3K4me3 and H3K27me3), DNA methylation and gene expression in any *Daphnia* species. We demonstrate the impact of the two histone modifications and DNA methylation individually, and more interestingly when they co-occur, on gene expression. Finally, this study provides further evidence in support of use of *Daphnia* as a model organism for research into epigenetic regulation of traits and phenotypic plasticity.

## Methods

### *Daphnia pulex maintenance and induction of males*

Cultures of *Daphnia pulex* Eloise Butler strain (genotypes EB31 and EB45, originally sampled from Eloise Butler pond in Minnesota, [59] were maintained in standard COMBO as previously described [30, 60, 61]. To induce male *Daphnia,* sexually mature individual female *Daphnia* were treated with the crustacean reproductive hormone, methyl (2E, 6E)-farnesoate (MF) at a final concentration of 400 nM. This concentration is sufficient to induce male *Daphnia* at 100% efficiency [16]. Due to the instability of MF, medium was changed daily to ensure consistent exposure. The first brood was discarded, and male neonates were collected from 2nd – 3rd broods. Female *Daphnia* used in the ‘omics studies were not exposed to MF. Similar to the male samples, neonates from 2nd-3rd broods were collected and used in this study. Female and male cultures were maintained separately.

### DNA and RNA extraction and sequencing

Genomic DNA and RNA were extracted from a pool of samples with a mixture of different ages (3, 8 and 15 days old) using MasterPure DNA purification kit (Epicentre, USA) and RNeasy Micro Kit (Qiagen Ltd., UK), respectively as described by Athanasio et al. 2016 and 2018 [61, 62]. DNA for the whole genome bisulfite sequencing (WGBS) was extracted from both genotypes (EB31 & EB45), from 3 female and 3 male *Daphnia* pools from each genotype. The ChIP-seq and RNA-seq samples were prepared from only one genotype (EB45). DNA for the ChIP-seq was extracted from 3 female, 3 male and 2 input control pools. RNA for the gene expression and splicing analysis was extracted from 2 female and 3 male *Daphnia* pools. The whole genome bisulfite sequencing (WGBS) libraries and the RNA sequencing libraries (RNA-seq) were prepared as described in our previous publication [30]. Briefly, the EpiGenome Methyl-Seq kit (Epicentre, USA) was used to prepare the WGBS libraries and sequenced (2x80bp) using Illumina NextSeq 500 platform at the Centre for Genomics and Bioinformatics, Indiana University. The RNA-seq libraries were prepared using the Illumina TruSeq standard mRNA kit and sequenced (1x85bp) using Illumina NextSeq 500 platform at the Centre for Genomics and Bioinformatics, Indiana University. The chromatin immunoprecipitation sequencing libraries (ChIP-seq) were prepared using the iDeal-seq kit, H3K4me3 (C15410003–50, 1 μg/reaction), H3K27me3 (C15410195, 1 μg/reaction) antibodies and sequenced using Illumina HiSeq 2500 (1 × 50 bp) as part of a service provided by Diagenode (Belgium). Briefly, *Daphnia* samples (30 mg wet tissue per sample) were homogenised in 1 ml of PBS/1%formaldehyde using Dounce homogenizer. The collected cells were lysed and the nuclei were collected and sonicated to a final size of 80–400 bp. The mentioned antibodies were used to prepare test samples according to the manual for the iDeal ChIP-seq kit. The IP samples and input samples were quantified using the Qubit dsDNA HS kit. Library preparation was performed on the IP and input samples using the MicroPLEX library preparation protocol on 500 pg of DNA. The amplified libraries (13 PCR cycles) were purified using AMPure beads, quantified using the Qubit ds DNA HS kit and analysed on Bioanalyzer. The prepared libraries were then sequenced on HiSeq 2500. This project has been deposited at NCBI GEO under accession GSE12442.

### Pre-processing, mapping, preliminary analysis

lllumina adapters (using core sequence: AGATCGGAAGAGC) and nucleotides with low quality (Phred score < 20) were removed with cutadapt (v.1.11) [63]. The filtered reads were mapped to the reference genome of *Daphnia pulex* PA42 (GCA_900092285.1) [34] using BWA Meth (v.0.10) [64] for bisulfite-treated DNA samples, BWA-MEM (v.0.7.15-r1140) [65] for the non-bisulfite treated DNA samples (ChIP-seq and reference DNA), and with RSEM (v.1.3.0) [66] using STAR aligner (v.2.5.3a) [67] for the RNA-seq samples, with default settings. The *Daphnia pulex* gene models used in the analysis are from November 2017 obtained from the arthropod database in eugenes (Genomic Information for Eukaryotic Organisms; http://arthropods.eugenes.org) produced by Don Gilbert using EvidentialGene [68].

### Analysis of gene expression and splicing data

Expression changes were analysed at gene and transcript levels using EBSeq (v.1.20.0) [69], with default settings. Genes and transcripts with significant expression difference between male and female *Daphnia* (with posterior probability of differential expression < 0.05) were analysed further. An additional alternative splicing analysis was conducted on the same filtered reads used for the expression analysis, using the de novo splicing predictor, KisSplice (v2.4.0-p1) [70] with default settings. The potential splicing events detect by KisSplice (type_1) were mapped back to the reference genome (GCA_900092285.1) with STAR aligner (v2.5.2a) [67], using default settings. The mapping results were analysed with KisSplice2RefGenome (v.1.0.0) [71] to identify the types of splicing events that occurred in the samples. Alternative splicing events were analysed for sex induced (male vs female) differential changes with kissDE (v1.5.0) [71]. Splicing events that did not map to known genes or mapped to multiple locations as well as events that were low coverage were excluded. Splicing events that were insertions, deletions or SNPs according to the genomic mapping were also removed.

### Analysis of DNA methylation data

Differential methylation analysis was done using methylKit (v.1.3.0) [72]. CpG sites with abnormally high (> 98 percentile) coverage were removed, as well as sites that were not covered in all samples or had zero methylated reads in more than half of the samples (*n* = 6/12). Logistic regression was used to analyse differential CpG methylation between male and female, using genotype (EB31 and EB45) as a co-variable. The Q-values were adjusted using the SLIM method [73].

### Analysis of chromatin immunoprecipitation sequencing data

The DNA fragments containing histone modification (H3K4me3 and H3K27me3) were purified, sequenced and aligned to the genome. The ChIP-seq reads were filtered by mapping quality (MAPQ > 30) to reduce background noise from unspecific mapping. The genomic locations where the DNA fragments were concentrated (peaks) were identified. The peaks corresponding to histone modifications (H3K4me3 and H3K27me3) were called with MACS2 (v.2.1.0.20151222) [74], separately for each sample without sifting model building using 132Mbp as an estimate of the mappable genome size and predicted fragment sizes 134 bp (for H3K4me3) and 144 bp (for H3K27me3) as estimated from the data. Differential analysis of histone peaks (narrowPeak) were achieved using DiffBind (v.2.8.0) [75], by comparing the male and female samples against each other (*n* = 3 for both sexes and histone modifications) and against the input controls (*n* = 2). The peaks for H3K27me3 were mapped to the nearest transcript, and the peaks for H3K4me3 were mapped against the nearest exon 1. Differential peaks (FDR < 0.05) within 200 bp of known transcripts (H3K27me3) or exon 1 (H3K4me3) were retained for further analysis.

### Enrichment analysis

The differentially regulated (FDR < 0.05) genes (containing CpG methylation, modified histones, expression or splicing changes) were analysed for enrichment in Reactome pathways [76] with ClusterProfiler (v.3.8.1) [77] and ReactomePA (v.1.24.0) [78]. Since *Daphnia pulex* genes are not annotated in Reactome, we used protein blast (with e-value <1e^− 20^) to identify orthologous genes in humans. The reference genes (universe) for the enrichment analysis were limited to only those human genes that were identified by blast and had NCBI gene IDs (9992 *Daphnia pulex* genes, matching to 6013 unique genes). 40% (4014) of these genes were annotated in the Reactome database.

## Supplementary information

**Additional file 1: Table S1.** Differentially expressed or regulated events between male and female *Daphnia pulex*. Results are in separate tabs; A) Differentially expressed transcripts, B) Differentially expressed genes, C) Differentially expressed splice variants, D) Differentially methylated CpGs, E) Differentially modified histone H3 trimethylated at lysine 4 (H3K4me3), F) Differentially modified histone H3 trimethylated at lysine 27 (H3K27me3).

**Additional file 2: Table S2**. Enriched Reactome pathways in differentially expressed or regulated events between male and female *Daphnia pulex*. A) Enrichment of differentially expressed transcripts between male and female *D. pulex*, B) Enrichment of differentially expressed genes, C) Enrichment of differentially expressed transcripts not contained in differentially expressed genes, D) Enrichment of differentially regulated spice variants between male and female, E) Enrichment of genes with differentially methylated CpGs, F) Enrichment of genes with differentially regulated histone H3 trimethylation at lysine 4 (H3K4me3), G) Enrichment of genes with differentially regulated histone H3 trimethylation at lysine 4 (H3K4me3), H) Enrichment analysis of genes with co-occurring modifications (CpG methylation, H3K4me3 and H3K27me3), I) Enrichment analysis of main clusters of genes with co-varying expression and modifications from heatmap in Fig. 7.d.

**Additional file 3: Fig. S1**. Comparison of sex-biased expression between *D. magna* and *D. pulex*. The orthologs were identified with blastp and limited to single gene matches with e-values <1e^− 20^. Sex-biased genes in *D. magna* are based on [26]. Genes marked in red are significantly different (PPEE< 0.05) in *D. pulex*, based on this study. *Doublesex* genes (*DapmaDsx1* and *DapmaDsx2*) are highlighted in purple.

## Data Availability

This project has been deposited at NCBI GEO under accession GSE12442. The reference genome and chromosomal assignment of scaffolds for *Daphnia pulex* is based on Ye et al. 2017 (DOI:10.1534/g3.116.038638). The *Daphnia pulex* gene models are from the arthropod database in eugenes (Genomic Information for Eukaryotic Organisms) produced by Don Gilbert using EvidentialGene (DOI: 10.1101/829184). Expression data for *Daphnia magna* sex-biased genes are from Molinier et al. 2018 (DOI:10.1534/g3.118.200174).

## Abbreviations

ChIP: Chromatin Immunoprecipitation
DMC: Differentially methylated CpGs
DSX: Doublesex gene
ESD: Environmental Sex Determination
FDR: False discovery rate
FPKM: Fragments per kilobase of transcript per million mapped reads
H3K27me3: Histone H3 trimethylated at lysine 27
H3K4me3: Histone H3 trimethylated at lysine 4
HSP: Heat Shock Proteins
MF: Methyl Farnesoate
WGBS: Whole genome bisulfite sequencing
