# Supplementary figures and images for: A comprehensive epigenomic analysis of phenotypically distinguishable, genetically identical female and male Daphnia pulex
Source: BMC Genomics. 2020 Jan 6;21:17. doi: 10.1186/s12864-019-6415-5 (PMC6945601; doi:10.1186/s12864-019-6415-5)

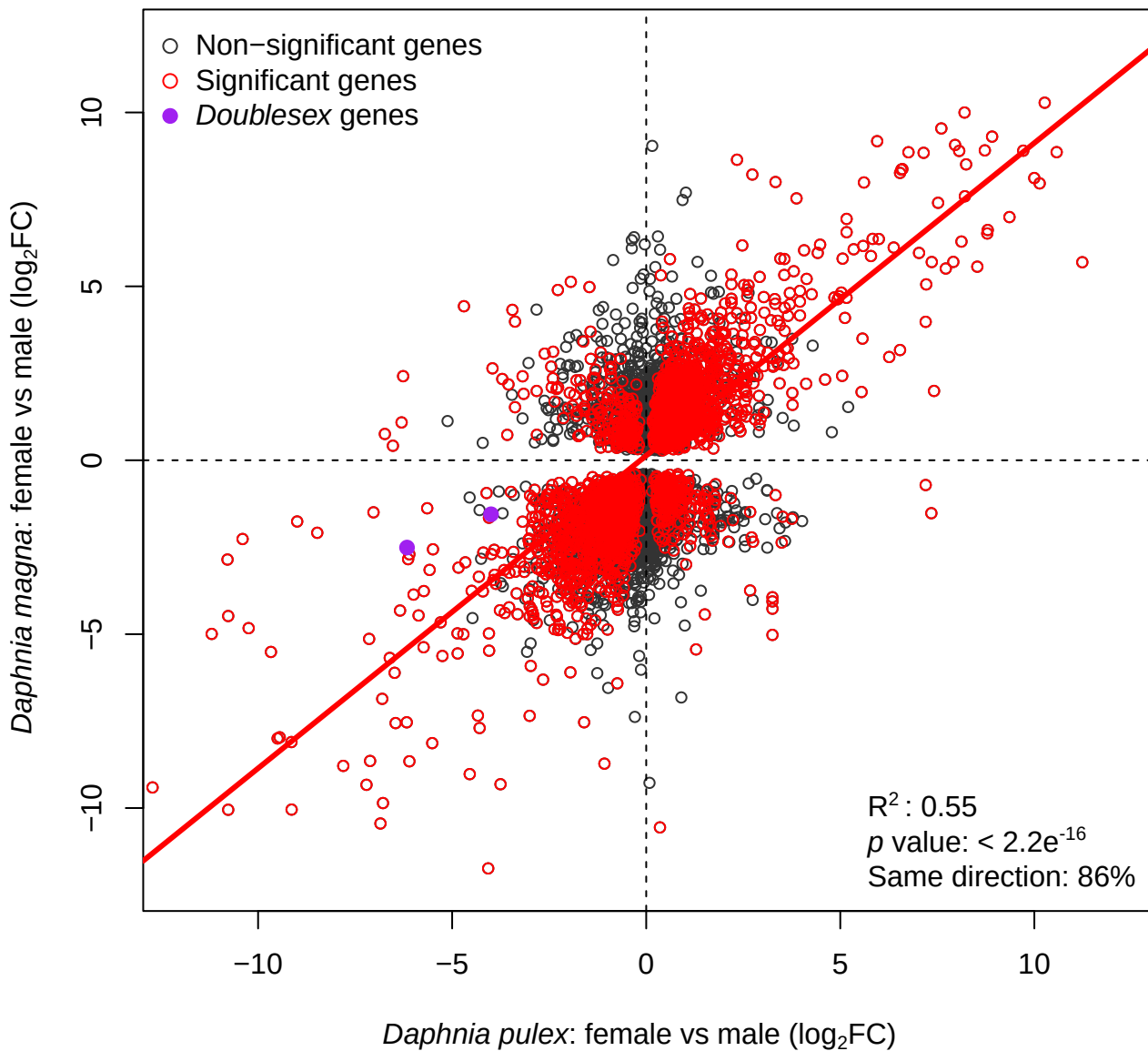

Supplement: Supplementary file 3 — Additional file 3: Fig. S1. Comparison of sex-biased expression between D. magna and D. pulex. The orthologs were identified with blastp and limited to single gene matches with e-values <1e− 20. Sex-biased genes in D. magna are based on [26]. Genes marked in red are significantly different (PPEE< 0.05) in D. pulex, based on this study. Doublesex genes (DapmaDsx1 and DapmaDsx2) are highlighted in purple. [file 12864_2019_6415_MOESM3_ESM.pdf]
